# Targeting Neuropilin-1 with Nanobodies Reduces Colorectal Carcinoma Development

**DOI:** 10.3390/cancers12123582

**Published:** 2020-11-30

**Authors:** Yannick De Vlaeminck, Stefano Bonelli, Robin Maximilian Awad, Maarten Dewilde, Sabrina Rizzolio, Quentin Lecocq, Evangelia Bolli, Ana Rita Santos, Damya Laoui, Steve Schoonooghe, Luca Tamagnone, Cleo Goyvaerts, Massimiliano Mazzone, Karine Breckpot, Jo A. Van Ginderachter

**Affiliations:** 1 Laboratory for Molecular and Cellular Therapy, Department of Biomedical Sciences, Vrije Universiteit Brussel, 1090 Brussels, Belgium; yannick.de.vlaeminck@vub.be (Y.D.V.); Robin.Maximilian.Awad@vub.be (R.M.A.); Quentin.Lecocq@vub.be (Q.L.); cleo.goyvaerts@vub.be (C.G.); 2 Laboratory for Cellular and Molecular Immunology, Vrije Universiteit Brussel, 1040 Brussels, Belgium; s.bonelli3@gmail.com (S.B.); evangeliabolli@gmail.com (E.B.); dlaoui@vub.be (D.L.); Steve.Schoonooghe@vub.ac.be (S.S.); 3 Myeloid Cell Immunology Lab, VIB Center for Inflammation Research, 1040 Brussels, Belgium; 4 VIB Discovery Sciences, 3000 Leuven, Belgium; maarten.dewilde@kuleuven.vib.be (M.D.); anarita.santos@kuleuven.vib.be (A.R.S.); 5 Candiolo Cancer Institute-FPO, IRCCS, 10060 Candiolo, Italy; sabrina.rizzolio@ircc.it; 6 Department of Life Sciences and Public Health, Università Cattolica del Sacro Cuore, 00100 Rome, Italy; lutamagn@gmail.com; 7 Department of Oncology, Fondazione Policlinico Universitario “A. Gemelli”, IRCCS, 00100 Rome, Italy; 8 Laboratory of Tumor Inflammation and Angiogenesis, VIB Center for Cancer Biology, 3000 Leuven, Belgium; massimiliano.mazzone@kuleuven.vib.be; 9 Department of Oncology, Laboratory of Tumor Inflammation and Angiogenesis, Center for Cancer Biology, KU Leuven, 3000 Leuven, Belgium

**Keywords:** neuropilin-1, nanobody, single-domain antibody fragment, cancer, immune checkpoint, immunotherapy, tumor-associated macrophage, semaphorin, plexin

## Abstract

**Simple Summary:**

Neuropilin-1 is a co-receptor for semaphorins and vascular endothelial growth factor family members. Neuropilin-1 can be expressed on tumor cells, tumor-infiltrating myeloid and lymphoid cells and has been linked to a tumor-promoting environment. We investigated nanobodies (Nbs) targeting neuropilin-1 for their potential to hamper colorectal carcinoma development in mice. Our data suggest that targeting neuropilin-1 in cancer using neuropilin-1 blocking Nbs delays tumor growth and extends the survival through a shift in the anti-tumor macrophage/pro-tumor macrophage ratio and activation of colorectal cancer-specific CD8^+^ T cells. These findings provide a rationale for the further development of Nbs targeting human neuropilin-1 and bringing them from the bench to the bedside.

**Abstract:**

Neuropilin-1 (NRP-1) is a co-receptor for semaphorins and vascular endothelial growth factor (VEGF) family members that can be expressed on cancer cells and tumor-infiltrating myeloid, endothelial and lymphoid cells. It has been linked to a tumor-promoting environment upon interaction with semaphorin 3A (Sema3A). Nanobodies (Nbs) targeting NRP-1 were generated for their potential to hamper the NRP-1/Sema3A interaction and their impact on colorectal carcinoma (CRC) development was evaluated in vivo through the generation of anti-NRP-1-producing CRC cells. We observed that tumor growth was significantly delayed and survival prolonged when the anti-NRP-1 Nbs were produced in vivo. We further analyzed the tumor microenvironment and observed that the pro-inflammatory MHC-II^high^/trophic MHC-II^low^ macrophage ratio was increased in tumors that produce anti-NRP-1 Nbs. This finding was corroborated by an increase in the expression of genes associated with MHC-II^high^ macrophages and a decrease in the expression of MHC-II^low^ macrophage-associated genes in the macrophage pool sorted from anti-NRP-1 Nb-producing tumors. Moreover, we observed a significantly higher percentage of tumor-associated antigen-specific CD8^+^ T cells in tumors producing anti-NRP-1 Nbs. These data demonstrate that an intratumoral expression of NRP-1/Sema3A blocking biologicals increases anti-tumor immunity.

## 1. Introduction

NRP-1 is a multifunctional, transmembrane, non-tyrosine kinase surface glycoprotein that has an important role in cancer and immunity [1]. NRP-1 is a co-receptor that binds several ligands, including VEGF and class III semaphorin family members, as such interacting with VEGF receptors and plexins, respectively. Expression of NRP-1 on cancer cells is correlated to their increased oncogenic activity, promoting cancer cell survival, inducing angiogenesis and contributing to therapy resistance [2]. 

In addition, NRP-1 expression has been described on various immune cells in the tumor microenvironment (TME), in particular on macrophages, dendritic cells (DCs) and regulatory T cells (Tregs) [3,4]. Expression of NRP-1 on these tumor-infiltrating immune cells has been linked to several functions, most of them associated with tumor promotion. In the case of tumor-associated macrophages (TAMs), NRP-1, together with PlexinA1/A4, acts as a guide for these cells to migrate to the hypoxic core of the tumor in response to semaphorin 3A (Sema3A), where they exert tumor-promoting functions [5]. In the hypoxic core, NRP-1 expression on TAMs is transcriptionally repressed, as such trapping these cells in those tumor regions where they suppress the activity of NRP-1^+^ CD8^+^ T cells [6] and stimulate Treg activity [5,7,8]. Genetically interrupting the migration of NRP-1-expressing TAMs to the hypoxic tumor core abrogates their acquisition of pro-tumor functions, instead allowing the induction of anti-tumor T helper 1 (T_H_1) and cytotoxic T lymphocyte (CTL) responses and a reduction of tumor progression [5,7,9]. In mice, NRP-1 is also considered a marker of Tregs, which is directly linked to their immunosuppressive activity and attraction to VEGF-rich tumors [10,11]. The absence of NRP-1 in CD4^+^ T cells led to the activation of an anti-tumor immune response in melanoma-bearing mice, as evidenced by an increase in CD8^+^ T cells and a decrease in Tregs within the TME. The net effect was reduced tumor burden and consequently improved survival in mice [11]. Another interesting observation is that NRP-1 deficiency in Tregs confers T_H_1 functions to these cells, including production of IFNγ, thereby influencing other Tregs and CD8^+^ T cells and stimulating anti-tumor immunity [12]. Moreover, it was shown that a reduction in IL-10 dampens NRP-1 signaling in Tregs and as such facilitates the adoption of T_H_1 and T_H_17-like functions [13].

Obviously, the link between NRP-1 expression on tumor-infiltrating immune cells and their tumor-promoting activity has instigated research in the development of NRP-1-blocking moieties, amongst others, monoclonal antibodies (mAbs) and tumor-penetrating peptides (TPPs). While mAbs that antagonize NRP-1 signaling have entered clinical testing, the outcome has been disappointing [14,15]. As the weak therapeutic efficacy is at least partly attributed to the inability of mAbs to penetrate solid tumors beyond a depth of 3–5 mm, alternative NRP-1-binding moieties with a smaller size have been developed, in particular TPPs [1]. These peptides have a cryptic C-terminal motif (RXXR/K) that is essential for binding to different receptors containing a CendR motif, including NRP-1. This motif is also used by VEGF and Sema3A to bind to NRP-1 [1,16]. However, the use of TPPs is hampered by non-specific cytolytic activity. Therefore, we set out to develop NRP-1-targeting nanobodies (Nbs), as these antigen-binding moieties, derived from camelid heavy chain only antibodies, are small in size (12–15 kDa), have a strong tumor-penetrating potential and their single-domain nature allows molecular engineering [17,18,19]. As a proof of concept, we engineered murine MC38 colon carcinoma cells to secrete anti-NRP1 Nbs and evaluated the Nbs’ ability to modulate the tumor microenvironment (TME) and to affect tumor growth.

## 2. Results

### 2.1. Generation and Characterization of NRP-1-Specific Nanobodies

**Figure 1:**
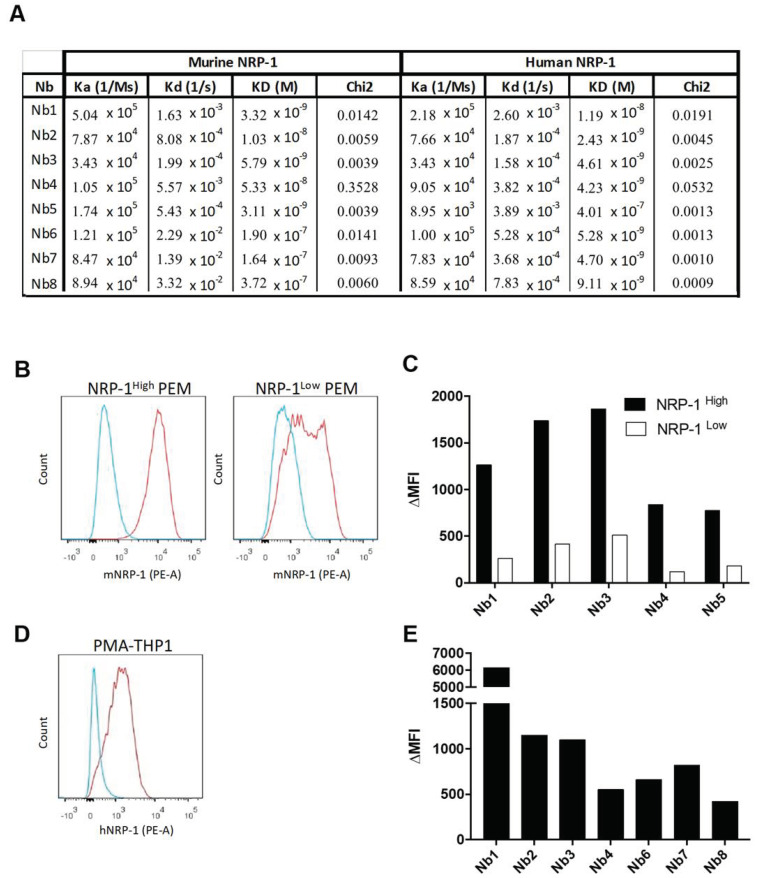
Selection and characterization of neuropilin-1 (NRP-1)-specific Nbs. (**A**) Association (Ka), dissociation (Kd) and equilibrium (K_D_) constants of the different nanobodies (Nbs) towards recombinant murine and human NRP-1 are extrapolated through bio-layer interferometry-based Octet. Model fitting chi-square values are also shown. (**B**) Expression of mouse NRP-1 (red line) on peritoneal macrophages (PEMs) isolated from *Nrp-1*^FL/FL^ mice (NRP-1^high^, left panel) and LysM^Cre^ *Nrp-1*^FL/FL^ mice (NRP-1^low^, right panel). The blue line represents the staining with an isotype-matched control antibody. The red line represents the staining with a phycoerythrine (PE)-conjugated NRP-1 mAb. (**C**) The graph shows the mean fluorescence intensity (ΔMFI) obtained after binding of the Nbs and their subsequent detection with an anti-HIS tag antibody on PEMs isolated from NRP-1^FL/FL^ mice (NRP-1^high^, black bar) and LysM^Cre^ *Nrp-1*^FL/FL^ mice (NRP-1^low^, white bar). (**D**) Expression of human NRP-1 (red line) on phorbol 12-myristate 13-acetate (PMA)-treated THP1 cells. The blue line represents the staining with an isotype-matched control antibody. The red line represents the staining with a PE-conjugated NRP-1 mAb. (**E**) The graph shows the ΔMFI obtained after binding of the Nbs and their subsequent detection with an anti-HIS tag antibody on PMA-treated THP1 cells. The ΔMFI was calculated as MFI (anti-NRP-1 + anti-HIS) minus MFI (isotype control) (*n* = 1).

An Nb phage display library was prepared from peripheral blood lymphocytes of an alpaca that was immunized with alternated injections of recombinant murine and human NRP-1 protein. This library was screened in an ELISA for Nbs that bind to recombinant human or mouse NRP-1, or both. This resulted in the selection of four potentially cross-reactive Nbs (Nb1, Nb2, Nb3 and Nb4), one mouse-specific binder (Nb5) and three human-specific (Nb6, Nb7 and Nb8) binders. None of the Nbs showed binding to human or mouse NRP-2, the closest homolog of NRP-1. Bio-layer interferometry measurements corroborated that Nb1, Nb2, Nb3 and Nb4 showed a high affinity for recombinant human and mouse NRP-1, while Nb6, Nb7 and Nb8 strongly bound to human NRP-1, and Nb5 to mouse NRP-1 (Figure 1A). We next assessed whether the selected Nbs recognized native NRP-1 at the cell surface. Flow cytometry showed that Nb1, Nb2, Nb3, Nb4 and Nb5 bound mouse NRP-1 expressed at the surface of thioglycolate-elicited peritoneal macrophages (PEMs, defined as CD11b^+^ F4/80^+^ Ly6G^−^, Siglec-F^−^ Ly6C^low^ cells) from NRP-1^WT^ mice. The reduction in NRP-1 expression at the surface of PEMs from LysM-cre x Nrp-1^f/f^ mice (conditional deletion of NRP-1 in macrophages and neutrophils), as determined via the use of an anti-mNRP1 mAb (Figure 1B), also led to a strongly reduced binding of Nb1, Nb2, Nb3, Nb4 and Nb5 (Figure 1C). Human THP1 monocytic leukemia cells that were treated with phorbol 12-myristate 13-acetate (PMA) also expressed NRP-1 (Figure 1D), shown via the use of an anti-hNRP-1 mAb, and also bound Nb1, Nb2, Nb3, Nb4, Nb6, Nb7 and Nb8 (Figure 1E). We continued the study with Nb1 based on the cross-reactivity and best binding capacity on human NRP-1. 

### 2.2. Anti-NRP-1 Nb1 Inhibits the NRP-1/Sema3A Interaction

**Figure 2:**
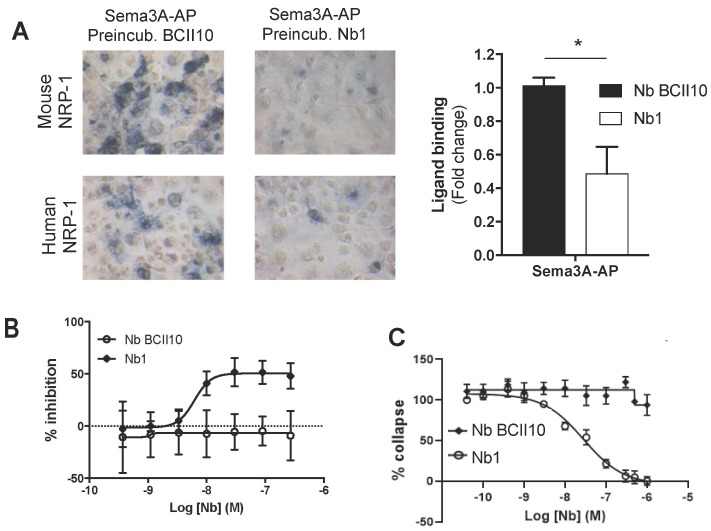
Nb1 interferes with the binding between NRP-1 and Sema3A. (**A**) The microscopic images show mNRP-1^+^ or hNRP-1^+^ COS cells preincubated with BCII10 or Nb1 and stained with p-nitrophenylphosphate after incubation with alkaline phosphatase (AP) conjugated Sema3A (20× magnification). The graph on the right shows the fold change in the binding of Sema3A to human NRP-1 in the presence of Nb1 (white bar) (*n* = 3). (**B**) The graphs show the result of the AlphaScreen on mouse NRP-1. The curve shows an increasing concentration of BCII10 (white dots) or Nb1 (black squares) competing with Sema3a-Fc for binding with biotinylated NRP-1 (*n* = 3). (**C**) The graph shows the percentage reduction in cell area (µm^3^) of human umbilical vein endothelial cells (HUVECs) treated for 30 min with an increasing concentration of Nb1 (black dots) or BCII10 (black squares), followed by a Sema3a-Fc incubation of 30 min (*n* = 3). Statistical analysis was performed by the Student’s *t*-test and asterisks represent a *p*-value < 0.05 (*).

Sema3A has been shown to promote tumor growth by attracting NRP-1^+^ TAMs to the hypoxic tumor core [5], suppressing the activity of NRP-1^+^ CD8^+^ T cells [6] and stimulating Treg activity [8]. To evaluate whether the cross-reactive anti-NRP-1 Nb1 was able to interfere with Sema3A binding, we incubated both hNRP-1^+^ and mNRP-1^+^ COS cells with alkaline phosphatase (AP)-conjugated Sema3A in the presence of Nb1 or the BCII10 control Nb [20]. Nb1 strongly reduced Sema3A binding to both mouse and human NRP-1 (Figure 2A). Importantly, the amplified luminescent proximity homogeneous assay (AlphaScreen™) corroborated this finding, again showing Nb1 interference with the binding of Sema3A to mouse and human NRP-1 (Figure 2B). To assess whether the Nb1-mediated NRP-1 blockade has physiological consequences, we studied the collapse (as measured by a reduction in total cell area) of NRP-1^+^ human umbilical vein endothelial cells (HUVECs) treated with Sema3A-Fc. While BCII10 did not prevent HUVEC collapse, Nb1 dose-dependently blocked this phenomenon (Figure 2C).

### 2.3. Anti-NRP-1 Nb1 Delays the Outgrowth of CRC Cells

We next studied whether Nb1 can affect tumor growth. The CRC model MC38 was chosen, as it has been used to study NRP-1 effects on Tregs [21] and because these tumors are highly infiltrated with myeloid cells, primarily macrophages, which may also express NRP-1 [22]. Moreover, MC38 cells showed marginal expression of NRP-1, excluding direct effects of Nb1 on cancer cell behavior. 

**Figure 3:**
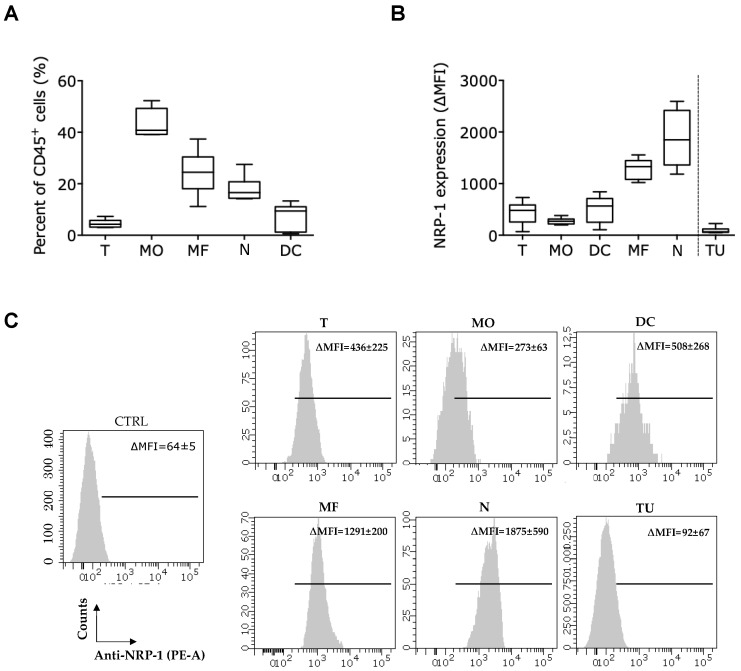
The colorectal carcinoma (CRC) model MC38 is characterized by a high infiltration with NRP-1^+^ tumor-infiltrating myeloid cells, while NRP-1 is expressed at low levels on cancer cells and T cells. (**A**,**B**) MC38 tumors were grown at the lower back of C57BL/6 mice up to a volume of 944 ± 237 mm^3^, after which tumors were isolated and reduced to single-cell suspensions. Expression of NRP-1 was evaluated on tumor-infiltrating immune cells and non-immune cells in flow cytometry. (**A**) The graph shows the percentage of T cells (T, CD45.2^+^ CD3^+^), monocytes (MO, CD45.2^+^ CD11b^+^ CD11c^−^ F4/80^−^ Ly6C^+^), macrophages (MF, CD45.2^+^ CD11b^+^ F4/80^+^ CD11c^−/low^ Ly6C^−^), neutrophils (N), CD45.2^+^ CD11b^+^ Ly6G^+^) and DCs (CD45.2^+^ CD11b^−^ CD11c^+^) that infiltrate MC38 tumors. The data are a summary of two independent experiments (*n* = 2, mice per condition (mpc) = 3). (**B**) The graph and **(C)** histograms show the expression of NRP-1 on the aforementioned immune cells and CD45^−^ non-immune cells (TU). The ∆MFI was calculated as the MFI of the anti-NRP-1 antibody minus the MFI of samples lacking the antibody staining NRP-1 (*n* = 2, mpc = 3).

The CD45^+^ immune infiltrate of MC38 tumors with a mean tumor volume of 944 ± 237 mm^3^ consisted of monocytes (43 ± 5%), macrophages (24 ± 8%), neutrophils (18 ± 5%), DCs (7 ± 5%) and T cells (4 ± 1%) (Figure 3A, gating strategy in Figures S1 and S2). These cells all expressed NRP-1 at their surface at varying levels, with neutrophils and macrophages showing the highest NRP-1 levels (Figure 3B,C). Notably, non-immune CD45^−^ cells (TU) from the MC38 tumor single cell suspension, including the cancer cells, hardly expressed NRP-1, suggesting that any intratumoral Nb1 effects are likely to be directed towards immune cells (Figure 3B,C). 

**Figure 4:**
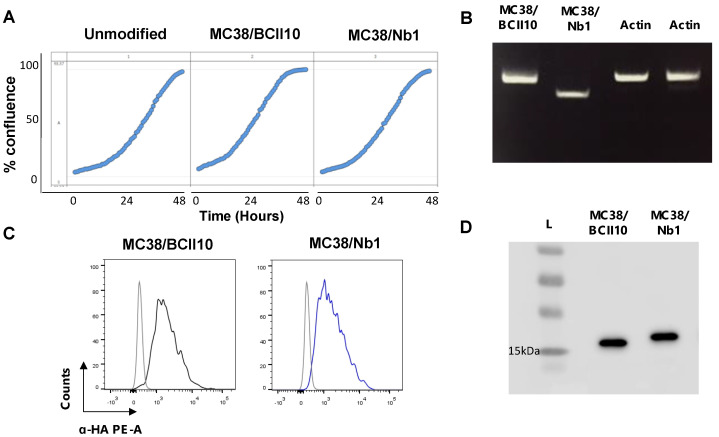
MC38/Nb1 and MC38/BCII10 show similar characteristics to unmodified MC38 cells and produce Nbs. (**A**) The graphs show the relative confluence for the growth of unmodified MC38 cells or MC38 cells lentivirally engineered to express BCII10 or Nb1, measured at several time points over a period of 2 days using the Incucyte instrument. (**B**) The image shows the result of the RT-PCR performed to detect expression of BCII10 or Nb1 mRNA in modified MC38 cells. (**C**) The histogram overlays show the intracellular staining of hemagglutinin (HA)-tagged Nbs in MC38/BCII10 (black line) or MC38/Nb1 (blue line). Cells stained with isotype-matched antibodies (gray histogram) served as a negative control. (**D**) Western blot was performed on supernatants collected from MC38/BCII10 or MC38/Nb1 to confirm secretion of the Nbs. The results shown in (**A**–**D**) are representative of three independent experiments (*n* = 3). Full western blot images of Figure 4B,D are available in the Supplementary Material (Figures S6 and S7).

Next, MC38 cells were lentivirally transduced with HA-tagged Nb1 or BCII10 coding sequences, to ensure a continuous, but local, production of these Nbs in the TME. Real-time analysis of the in vitro growth kinetics of MC38 cells did not show differences between the parental MC38 cells and their lentivirally transduced derivatives, referred to as MC38/Nb1 and MC38/BCII10 (Figure 4A). Transduced cells were confirmed to express Nb1 or BCII10 mRNA using RT-PCR, to produce Nbs as detected via intracellular flow cytometry and to secrete these Nbs in the supernatant, as shown via western blot (Figure 4B–D). 

**Figure 5:**
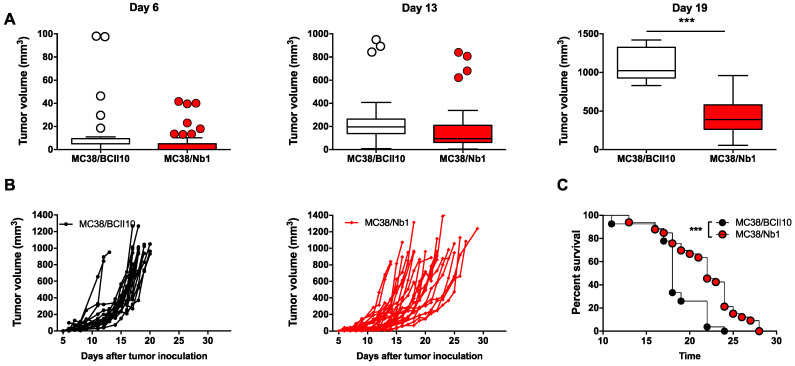
Local production of Nb1 delays MC38 tumor outgrowth in vivo. (**A**–**C**) MC38/BCII10 or MC38/Nb1 were grown at the lower back of C57BL/6 mice. Growth of MC38 tumors was monitored on a daily basis. (**A**) The box and whisker graphs show the range, mean and standard deviation of the tumor volume on days 6, 13 and 19. (**B**) The graphs show the tumor growth in each individual mouse. (**C**) Mice were killed when tumors reached a volume of 993 ± 115 mm^3^. The time to reach this tumor size was plotted in a Kaplan–Meier curve and the log-rank test was used to determine *p*-values. (**A**–**C**) The graphs summarize the results of three independent experiments (mpc = 23 for BCII10 and 28 for Nb1). Statistical analysis was performed by the Student’s *t*-test for tumor growth and the log-rank (Mantel–Cox) test for the survival. Asterisks represent *p* < 0.001 (***).

Nb-secreting MC38 cells were then inoculated subcutaneously at the lower back of C57BL/6 mice and tumor growth was monitored. No significant differences were observed between MC38/Nb1 and MC38/BCII10 in the first two weeks of tumor growth. Interestingly however, by week three, the MC38/Nb1 tumor volume (436 ± 237 mm^3^) was significantly lower than that of MC38/BCII10 (1077 ± 215 mm^3^) (Figure 5A,B). In line with these findings, the survival was extended in mice bearing MC38/Nb1 tumors (median survival of 22 versus 18 days) (Figure 5C).

### 2.4. Presence of the Nb1 Anti-NRP-1 Nb in the TME of CRC Tumors Favors More Pro-Inflammatory Macrophages

**Figure 6:**
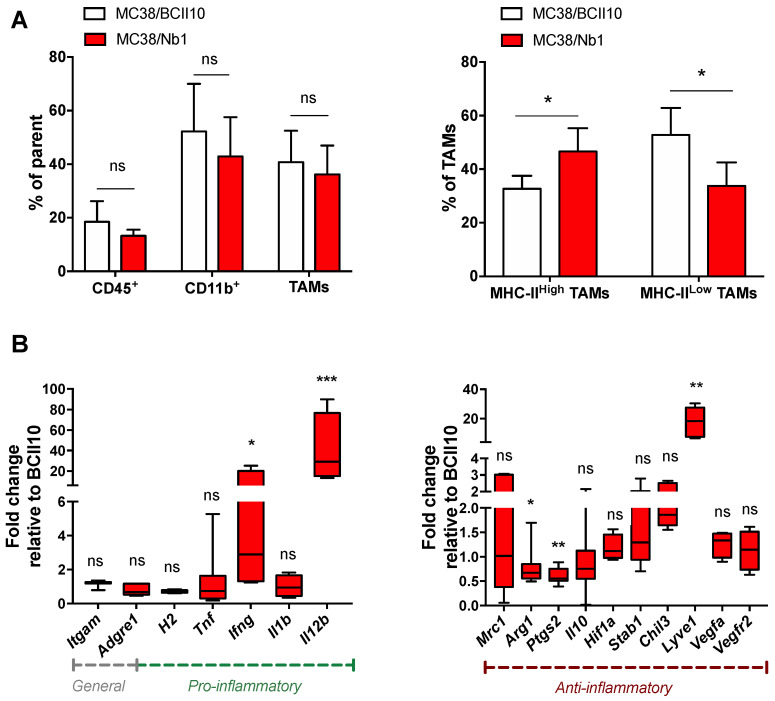
Production of Nb1 in the tumor microenvironment (TME) of MC38 tumors alters macrophage numbers and function. (**A**,**B**) MC38/BCII10 or MC38/Nb1 tumors were grown at the lower back of C57BL/6 mice. Growth of MC38 tumors was monitored on a daily basis. Tumors with a size of 458 ± 40 mm^3^ were isolated and reduced to single-cell suspensions. (**A**) Flow cytometry was used to determine the percentage of immune cells (CD45.2^+^), and within these immune cells, the percentage of myeloid cells (CD45.2^+^ CD11b^+^), and within these myeloid cells, the percentage of macrophages (CD45.2^+^ CD11b^+^ Ly6G^−^ Ly6C^−/low^ F4/80^+^). Macrophages were further subdivided into MHC-II^low^ or MHC-II^high^ cells. The bar graphs show the percentage of cells and summarize the results of two independent experiments (*n* = 2, mpc = 6). Statistical analysis was performed by the Student’s *t*-test and asterisks represent a *p*-value < 0.05 (*). (**B**) mRNA was isolated from macrophages sorted from tumors as CD45.2^+^ CD11b^+^ Ly6G^−^ Ly6C^−/low^ and F4/80^+^ cells. RT-qPCR was performed to determine the expression of *Itgam* (CD11b), *Adgre1* (F4/80), *H2* (MHC-II), *Tnf*, *Ifng*, *Il1b*, *Il12b*, *Mrc1* (CD206), *Arg1*, *Ptgs1* (Cox2), *Il10*, *Hif1a*, *Stab1*, *Chil3* (*Ym1*), *Lyve1*, *Vegfa* and *Vegfr2*. The box and whisker graph shows the range, mean and standard deviation of the fold in- or decrease in expression of the evaluated markers. The box and whisker graph summarizes the results of two independent experiments (*n* = 2, mpc = 6). Statistical analysis was performed by the Student’s *t*-test and asterisks represents *p* < 0.05 (*), *p* < 0.01 (**) and *p* < 0.001 (***).

We next assessed the impact of Nb1 on the immune contexture of MC38 tumors. Flow cytometry showed no significant changes in the percentage of neutrophils, monocytes, DCs or macrophages in MC38/Nb1 versus MC38/BCII10 tumors (Figure S3). However, we observed a change in the macrophage phenotype, with a significantly increased presence of MHC-II^high^ TAMs and significantly fewer MHC-II^low^ TAMs in MC38/Nb1 tumors (Figure 6A). MHC-II^high^ TAMs were reported to be more inflammatory/immune permissive [23,24]. To assess the inflammatory status of the TAMs, MHC-II^high^ and MHC-II^low^ TAMs were sorted from both tumor types and the expression of prototypical pro- or anti-inflammatory genes was tested. Expression of the pro-inflammatory cytokines *Ifng* and especially *Il12b* was significantly induced in TAMs from MC38/Nb1 tumors compared to MC38/BCII10 tumors, while the anti-inflammatory cytokine *Il10* did not change. A high *Il12b/Il10* balance is indicative of the pro-inflammatory status of macrophages [25,26]. In line with the inflammatory status of TAMs from MC38/Nb1 tumors, *Arg1* and *Ptgs2* expression, both associated with type 2 immune responses, were significantly reduced (Figure 6B). A notable exception is the upregulation of *Lyve1* in MC38/Nb1 TAMs, a marker typically associated with anti-inflammatory macrophages. Sema3A-stimulated migration of bone marrow-derived macrophages (BMDMs), which served as a surrogate for TAMs, was studied in vitro, showing reduced migration of BMDMs to Sema3A in the presence of Nb1, but not BCII10 (Figure S5).

### 2.5. Presence of the Nb1 Anti-NRP-1 Nb in the TME of CRC Tumors Favors Anti-Tumor T-Cell Responses

**Figure 7:**
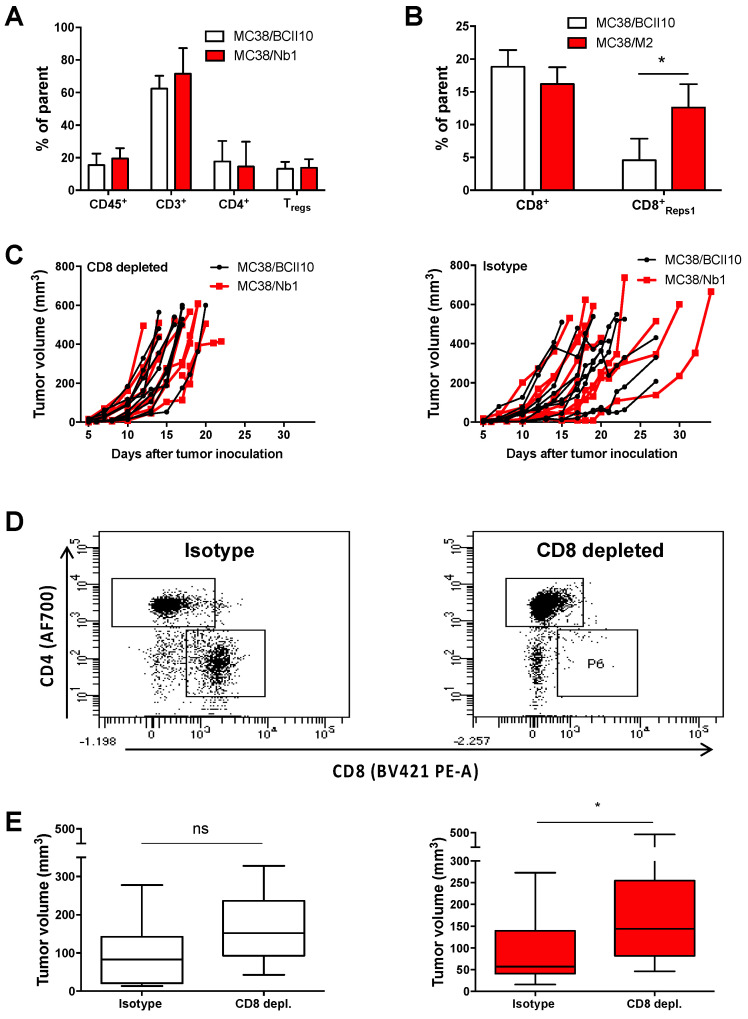
Production of Nb1 in the TME of MC38 tumors increases the percentage of tumor-specific CD8^+^ T cells. (**A**,**B**) MC38/BCII10 or MC38/Nb1 cells were subcutaneously injected in C57BL/6 mice. Growth of MC38 tumors was monitored on a daily basis. Tumors with a size of 534 ± 68 mm^3^ were isolated, reduced to single-cell suspensions and subjected to flow cytometry analysis. The bar graphs show the percentage of immune cells (CD45.2^+^) and, within these immune cells, the percentage of CD3^+^ T cells, which are further subdivided in CD4^+^ and CD8^+^ T cells. Within the CD4^+^ T cells, we further analyzed Tregs (CD25^high^ CD127^−^). Within the CD8^+^ T cells, Reps1-specific cells were analyzed. The bar graphs summarize the results as mean ±SEM of two independent experiments (*n* = 2, mpc = 10). Statistical analysis was performed by the Student’s *t*-test, and asterisks represent a *p*-value < 0.1 (*). (**C**–**E**) MC38/BCII10 or MC38/Nb1 cells were injected subcutaneously in C57BL/6 mice. Mice were injected intraperitoneally with 50 µg CD8-depleting or isotype-matched antibodies before tumor cell inoculation. Growth of MC38 tumors was monitored on a daily basis. (**D**) Depletion of CD8^+^ T cells was confirmed on splenocytes using flow cytometry. The dot plots show the presence of CD4^+^ and CD8^+^ T cells in mice treated with an isotype-matched control antibody, while only CD4^+^ T cells were detected in mice treated with CD8-depleting antibodies. The dot plots shown are representative of two experiments (*n* = 2, mpc = 5). (**E**) The box graphs show the range, mean and standard deviation of the tumor sizes on day 13 in the presence or absence of CD8^+^ T cells in MC38/BCII10 or MC38/Nb1 tumors. The box and whisker graph summarizes the results of two independent experiments (*n* = 2, mpc = 5). Statistical analysis was performed by the Student’s *t*-test and asterisks represent a *p*-value < 0.05 (*).

Finally, we assessed whether the reduced growth of MC38/Nb1 tumors could be correlated to differences in the intratumoral T cell compartment. No differences in the percentage of total CD3^+^ T cells, CD4^+^ T cells, CD4^+^ Tregs (identified as CD25^+^ CD127^−^) or CD8^+^ T cells were observed between MC38/Nb1 tumors and MC38/BCII10 tumors (Figure 7A, Figure S4). Nevertheless, a significantly higher percentage of Reps1 (MC38 neo-antigen)-reactive CD8^+^ T cells was present within MC38/Nb1 (12.6 ± 3.6%) compared to MC38/BCII10 (4.6 ± 3.3%) tumors, suggestive of an enhanced anti-tumor CD8^+^ T cell response (Figure 7B). To assess this point, we depleted CD8^+^ T cells using anti-CD8 antibodies and measured the growth of MC38/BCII10 and MC38/Nb1 tumors (Figure 7C). Complete systemic CD8^+^ T cell depletion was confirmed at the end stage of the experiment (day 17) (Figure 7D). In the absence of CD8^+^ T cells, MC38/Nb1 tumors grew significantly faster, comparable to the MC38/BCII10 tumors (Figure 7E). These data show that the intratumoral presence of anti-NRP1 Nbs is able to augment CD8^+^ T cell-dependent anti-tumor immunity.

## 3. Discussion

NRP-1 is expressed by various cells, including immune cells and cancer cells, and acts as a co-receptor for a variety of receptors implicated in cancer-promoting activities [1]. Therefore, NRP-1 has been put forward as an interesting target to manage cancer. Several strategies have been studied in preclinical tumor models to interfere with NRP-1 in the TME, ranging from genetic strategies, such as modification of cancer cells to express soluble NRP-1 or downregulate NRP-1 [27,28,29,30,31,32,33,34], to the use of mAbs that block NRP-1 [6,35,36,37,38,39]. These studies confirmed the tumor-promoting role of NRP-1 and showed that the targeting of NRP-1 can act directly on cancer cells or indirectly by modulating angiogenesis or immune cells in the TME, including Tregs, macrophages and CD8^+^ T cells [2,5,6,34,39]. For example, a macrophage-specific conditional deletion of NRP-1 resulted in smaller tumors and fewer pulmonary metastases in a subcutaneous Lewis lung cancer model [5].

A recent phase Ib study showed that the anti-NRP-1 mAb MNRP1685A elicited thrombocytopenia [15,40]. This toxic effect was caused by the mAb’s Fc-region that resulted in platelet consumption by inducing Fc-dependent platelet activation and binding to activated endothelial cells [40].

Another downside of the large size of mAbs (±150 kDa) is their inability to penetrate solid tumors efficiently, leaving part of the tumor untouched. Nbs may solve some of these limitations. Similar to mAbs, Nbs are characterized by high specificity and affinity for their target. However, Nbs are small (±15 kDa), and hence penetrate deep into tumors and even immunological synapses [41,42], while being rapidly cleared when unbound. It is this property that led to the evaluation of Nbs as therapy agents in the immuno-oncology field [43].

We generated Nbs targeting NRP-1 by immunizing an alpaca with human and mouse NRP-1 protein, which both include two coagulation factor V/VIII homology-like domains (b1 and b2) and two complement-binding-like (CUB) domains (a1 and a2). The mouse recombinant NRP-1 protein further included the meprin, A-5 protein and mu (MAM) domain. The MAM domain mediates dimerization of NRP-1, while the a1/a2 and b1/b2 domains aid binding to class III semaphorins and VEGF proteins, respectively [44]. From these, we selected Nb1 for in vivo analysis of its functional NRP-1 blocking capacity. This choice was based on the observation that Nb1 was able to bind human and mouse NRP-1 when analyzed using bio-layer interferometry-based Octet and interferes with the binding of Sema3A to NRP-1. 

Using a gene-based strategy to ensure the continuous production of Nb1 in the TME, we showed that NRP-1 blockade using Nb1 resulted in a delayed outgrowth of MC38 tumors and consequently enhanced survival, thereby confirming the importance of NRP-1 as a cancer target. The delay in tumor growth upon Nb1-mediated blockade of NRP-1 only became apparent in later stages of tumor growth (week 3) when the TME is established. This contrasts with several studies in which NRP-1 blockade showed differences in tumor growth already in the early stages of tumor development [11,45]. A main difference could be the lack of NRP-1 expression on the MC38 cancer cells, suggesting that the anti-tumor effect in this model is due to the blockade of NRP-1 on immune cells, of which myeloid cells showed the highest NRP-1 expression in MC38 tumors. 

When studying the TME to unravel the mechanisms that are at the basis of delayed tumor growth, we did not observe significant differences in the number of CD45^+^ immune cells or within the total number of CD11b^+^ or CD3^+^ cells. However, when focusing on CD11b^+^ myeloid cells, we observed that tumors producing Nb1 were enriched in MHC-II^high^ macrophages, correlating with enhanced expression of *Il12b* and *Ifng* and reduced expression of *Arg1* and *Ptgs2* (Cox2) in the TAM compartment. NRP-1 is not required for the recruitment of monocytes, which can then differentiate into macrophages, explaining why macrophage numbers are not affected by Nb1-mediated NRP-1 blockade [5]. However, NRP-1 controls the migration of TAMs to the hypoxic areas [5]. Once trapped in those areas, macrophages shift from an inflammatory anti-tumor to a pro-tumor phenotype, exerting several functions, such as T cell inhibition by depletion of L-arginine via arginase-1, and promotion of angiogenesis by secreting VEGF-A [46,47,48,49,50]. Hence, the TAM gene expression profile in MC38/Nb1 tumors could be consistent with a preferential localization outside of the hypoxic areas. Unexpectedly, the expression of the lymphatic vessel endothelial hyaluronan receptor (*Lyve1*), which was reported before to associate with alternatively activated macrophages, was significantly increased in macrophages present in Nb1-producing tumors [51]. The exact biological effect of the Lyve-1/hyaluronic acid interaction in myeloid cells has not yet been clarified [51], but, in various tumor types, Lyve-1^+^ macrophages have been primarily found in the marginal zone and not the central hypoxic region [52]. It is therefore tempting to speculate that the high expression of *lyve-1* in TAMs is an extra confirmation that anti-NRP-1 Nbs prevent their migration towards hypoxic areas. 

The increased presence of MHC-II^high^ macrophages, together with enhanced *Il12b* and *Ifng* expression, suggests favorable conditions for the induction of anti-tumor T cell activity in MC38/Nb1 tumors. Moreover, we observed that T cells in the TME of MC38 tumors showed low NRP-1 expression levels. This appears to be especially relevant in the case of anti-tumor CD8^+^ T cells, in which NRP-1 limits the development of long-term memory cells [53]. Hence, blocking anti-NRP-1 Nbs may directly and indirectly support anti-tumor T cells. Although we did not see an overall increase in CD4^+^ or CD8^+^ T cells in the MC38/Nb1 TME, there was a significant expansion of CD8^+^ T cells recognizing the Reps1 peptide, derived from an MC38 neo-antigen. A similar finding was recently published by Leclerc et al. [6], who showed in a B16F10 melanoma model that anti-NRP-1 mAbs enhanced the migration and cytotoxicity of CD8^+^ tumor-infiltrating T cells. When MC38/Nb1 tumors were grown in CD8 T cell-depleted mice, tumor growth significantly increased and became indistinguishable from MC38/BCII10 control tumors, illustrating that anti-NRP-1 Nbs unleashed an efficient anti-tumor CTL response.

In conclusion, our data demonstrate that targeting NRP-1 in cancer, using antagonistic anti-NRP-1 Nbs that prevent the interaction with Sema3A, delays growth of CRC tumors and extends the survival through a shift in the MHC-II^high^/MHC-II^low^ ratio and an activation of CRC-specific CD8^+^ T cells. These findings provide a rationale for the further development of Nbs targeting human NRP-1 and bringing them from the bench to the bedside.

## 4. Materials and Methods

### 4.1. Nb Generation

An alpaca (*Vicugna pacos*) was injected weekly for 6 weeks, alternating with 100 µg recombinant human (Phe_22_-Lys_64_4, NP_001019799) or mouse (Phe_22_-Pro_856_, NP_032763) NRP-1 protein (R&D Systems, Minneapolis, MN, USA) mixed with Gerbu LQ3000 (Gerbu Biotechnik). The last injection contained human and mouse NRP-1. Peripheral blood was collected, and lymphocyte mRNA was reverse transcribed to cDNA. Variable domain of heavy chain antibodycoding sequences were amplified and ligated onto a variant of a pHEN4 phagemid vector (pMECS). Using M13K07 helper phages, the Nb library was expressed on phages, and specific Nb phages were enriched by three rounds of selection on microtiter plates (Nunc, Sigma-Aldrich, Darmstadt, Germany) coated with recombinant NRP-1. Three separate enrichments were performed using: (1) only recombinant mouse NRP-1, (2) only recombinant human NRP-1, (3) alternately mouse and human recombinant NRP-1. An additional enrichment was performed using recombinant human NRP-1 but, this time, phages were eluted using 100 nM recombinant mouse Sema3A. Individual colonies were cultured and Nbs expressed and further screened through an ELISA for antigen recognition. NRP-1-specific Nbs were sequenced before being cloned into the vector pHEN6c (to encode a C-terminal His_6_ tag). Periplasmic expression and purification of Nbs was carried out as described [54]. BCII10, a Nb specific for bacterial β-lactamase, was used as control [20].

### 4.2. Bio-Layer Interferometry on the Octet^®^

Association, dissociation and equilibrium constants from purified Nbs as well as Nb1/Sema3A-Fc/NRP-1 binding competition was evaluated by bio-layer interferometry on the Octet RED96 system (FortéBIO, Fremont, CA, USA). Mouse or human recombinant NRP-1 proteins were coated on anti-steptavidin mouse IgG Fc capture (AMC) biosensors (FortéBIO) in 10 mM HEPES pH 7.5, 150 mM NaCl, 3 mM EDTA, 0.1% BSA, 0.05% Tween-20. Biotinylated hNRP-1 (R&D systems) or mNRP-1 (R&D systems) at 1 µg/mL was immobilized on the AMC biosensors to a signal of 1.5 nm. The association of the Nb was measured for 400 s, tailed by a dissociation experiment for 1200 s at 30 °C. Data were double reference-subtracted and aligned to each other in Octet Data Analysis software v9.0 (FortéBIO) based on a baseline measurement of blank control. Association and dissociation of non-saturated curves were fit in a global 1:1 model.

### 4.3. Cell Culture

The human HEK 293T, monkey COS and mouse MC38 colorectal carcinoma cells were obtained from the ATCC and were cultured in DMEM (Sigma-Aldrich) supplemented with 10% FBS (Tico Europe, Amstelveen, The Netherlands), 100 µg/mL penicillin, 100 µg/mL streptomycin and 2 mM L-glutamine (Sigma-Aldrich) (referred to as DMEM^+^). The human THP1 cells and HUVECs were obtained from ATCC and cultured in RPMI-1640 supplemented with 10% FBS, 100 µg/mL penicillin, 100 µg/mL streptomycin, 2 mM L-glutamine, 50 pM beta-mercaptoethanol (Sigma-Aldrich) and incubated for 24 h with 150 nM phorbol 12-myristate 13-acetate (PMA; Sigma) for macrophage differentiation. HUVECs were cultured in endothelial cell growth medium (EGM™-2) Bulletkit™ containing basal medium, supplements and growth factors (Lonza, Basel, Switzerland).

### 4.4. PEM Isolation 

Peritoneal exudate macrophages (PEMs) were collected by rinsing the peritoneal cavity of a C57BL/6 *Nrp-1*^FL/FL^ mouse with 5 mL cold PBS 4 days after 3 mL thioglycolate broth injection. Collected samples were washed in PBS by a 5-min centrifugation step at a relative centrifugal force of 453.

### 4.5. Sema3A Cell-Binding Assay 

The Sema3A cell-binding assay was described elsewhere [5]. Briefly, COS cells were transfected with mouse or human NRP-1 expression constructs. For a quantitative assessment of ligand binding in the presence of unlabeled competitor Nb1 or BCII10, cell-bound AP was revealed by incubation with the chromogenic soluble AP substrate p-nitrophenylphosphate (Sigma) and evaluated by spectrophotometry at 405 nm.

### 4.6. Mouse NRP1/Sema3A Competition AlphaScreen

Five microliters of biotinylated mouse NRP1 (R&D Systems, biotinylated in-house, final concentration 1.6 nM) were incubated with 5 µL of a dilution series of Nb1 for 1 h at room temperature. After 1 h, 5 µL of human Sema3A-Fc (R&D Systems, final concentration 6 nM) were added and incubated for an additional hour. Next, a mixture of 5 µL of 1/50 diluted AlphaScreen streptavidin donor beads and 5 µL of 1/50 Anti-human IgG (Fc specific) AlphaLISA Acceptor Beads was added, After 1 h, the AlphaCounts were measured according to the manufacturer’s instructions on an Envision multimode microplate reader (PerkinElmer).

### 4.7. HUVEC Collapse Assay

HUVECs where seeded at 4000 cells/well of a human fibronectin-coated 96-well plate in endothelial cell growth medium (ECGM) medium (C-22010 (Bioconnect, TE Huissen, The Netherlands). After 6 h, the HUVEC medium was replaced by DMEM supplemented with 10 % FBS. After 24 h, a dilution series of the Nbs was added (0.04–1000 nM) and incubated for 30 min, followed by a 20 min incubation with 8.8 nM Sema3A-Fc (R&D systems). Cells were fixed in 4% paraformaldehyde (PFA) and stained with phalloidin-488 and Hoechst (Thermo Fisher Scientific, Waltham, MA, USA). Imaging and analysis were performed with the Opera Phenix High Content Screening System (10× air magnification) (PerkinElmer, Waltham, MA, USA) according to the manufacturer’s protocol.

### 4.8. Mouse Bone Marrow-Derived Macrophages (BMDMs)

BMDMs were generated as described previously [55]. Bone marrow cells were flushed from tibiae and femurs of 8–12-week-old C57BL/6 mice using PBS. The cells were passed through a 40 µM cell strainer and centrifuged for 5 min at 453 rcf. The cell pellet was resuspended in Tris-buffered ammonium chloride for lysis of red blood cells. The cells were washed twice in PBS by a 5 min centrifugation step at 453 rcf. Bone marrow cells were cultured in 10 mm Petri dishes for 3 days at 5 × 10^6^ bone marrow cells in 10 mL DMEM+ (DMEM with 10% heat-inactivated FBS, 2 mM L-glutamine, 100 U/mL penicillin) and 50 ng/mL macrophage colony-stimulating factor (M-CSF, Immunotools). On day 3, 5 mL medium containing 50 ng/mL M-CSF were added. Cells were harvested on day 7.

### 4.9. Fluoroblok^TM^ BMDM Migration Assay

BMDMs were labeled with a 5 μM concentration of Calcein AM Dye for 30 min using the manufacturer’s protocol. For cell invasion experiments, prior to cell dispensing, inserts of 96-well FluoroBlok™ plates with 3 µM pores (BioTek, Agilent, Winooski, VT, USA) were rehydrated with warm PBS for 2 h in a humidified, 37 °C, ambient atmosphere incubator. After this hydration step, the basolateral chambers were loaded with DMEM+ containing Sema3A (100 ng/mL). Wells without Sema3A were used to study migration of BMDMs when unstimulated. The insert contained DMEM+ and 1 × 10^4^ BMDMs. Nb1 or BCII10 were added (100 µg/mL) to study their effect on BMDM migration. The loaded FluoroBlok™ plates were placed into the EnSight™ (PerkinElmer) at 37 °C and bottom fluorescence levels were measured across time. The slope of the curve was used to compare migration in response to Sema3A in the presence of Nb1 and BCII10.

### 4.10. Lentiviral Vector Production and Characterization 

The packaging plasmid pCMVΔR8.9 and VSV.G-encoding plasmid pMD.G were a gift from Dr. D. Trono (University of Geneva, Geneva, Switzerland). The transfer plasmid pHR’ trip CMV SIN [56] encoding BCII10 or Nb1, containing a murine IgK secretion signal (IgK) and hemagglutinin (HA) detection tag, was designed in silico using Snapgene^®^ and a gBlock^®^ Gene fragment was synthetized by Integrated DNA Technologies, Inc. (IDT, Leuven, Belgium). The sequence encoding BCII10 or Nb1 contained a pHR’ trip CMV SIN complementary overhang and was ligated into the pHR’ plasmid using the Gibson assembly method. The resulting vectors were transformed with the TransformAid Bacterial Transformation Kit (Thermo Fisher Scientific) in Neb5alpha *Escherichia coli* (*E. coli*) bacteria allowing the selection of recombinant clones on ampicillin-enriched agar plates.

Lentiviral vector production has been previously described [56,57]. In brief, HEK 293T cells were seeded and transfected using polyethyleneimine (PEI) (2 µg for each µg DNA, Polysciences Inc, Eppelheim, Germany) with 15, 30 and 45 µg of the pMD.G, pCMVΔR8.9 and pHR’ plasmid. The supernatant containing lentiviral vectors was harvested 48 and 72 h after transfection and concentrated 1000-fold by ultracentrifugation in a Beckman SW28 rotor (Optima LE-80K ultracentrifuge; Beckman Coulter, Palo Alto, CA, USA). The lentiviral vectors were titrated by seeding 10^5^ HEK 293T cells the day before transduction. The cells were either not transduced or transduced with the 1/500, 1/5000, 1/10,000 or 1/50,000 dilution of the lentiviral vector stock. After 72 h, cells were incubated with Brefeldin A (BD Biosciences, Erembodegem, Belgium) for 5 h to prevent secretion of the HA-tagged Nbs. Intracellular localized Nbs were visualized with a phycoerythrin (PE)-conjugated anti-HA polyclonal antibody (AbCam, Cambridge, UK) after permeabilization and fixation with BD Cytofix/Cytoperm according to the manufacturer’s protocol (BD Biosciences). The percentages of HA tag-positive cells were used to calculate the average amount of transducing units (TUs) per µL lentiviral vector stock using the following equation: (number of transduced cells × percent of transduced cells)/(volume used for transduction × 100) × factor of the dilution used.

### 4.11. Generation and Quality Control of Nb-Expressing MC38 Tumor Cells

MC38 cells (2 × 10^4^) were seeded in a flat-bottom 24-well plate in 200 µL DMEM^+^. These were transduced in the presence of 10 µg/mL protamine sulphate with lentiviral vectors (LVs) encoding BCII10 or Nb1 (MOI ranging from 1 to 10). Transduction was evaluated 72 h later in flow cytometry. MC38 cells with equal levels of Nb1 and BCII10 expression were expanded and transferred to a 6-well plate. Confluency was monitored for 48 h using Incucyte real-time analysis. Total RNA was extracted with the AllPrep DNA/RNA Mini Kit (Qiagen, Hilden, Germany) for RT-PCR to verify Nb expression using a primer blend with a common and Nb1 or BCII10-specific primer (Table 1). Western blot was performed on supernatant (20 µL) from stable Nb-expressing MC38 cells cultured at 80% confluency for 48 h to verify Nb secretion. Supernatant was loaded on a 10% SDS gel, transferred to a nitrocellulose membrane (GE Healthcare) and Nbs were detected with an anti-HA tag antibody that is conjugated to horseradish peroxidase (Abcam, Cambridge, UK). The chemiluminescent signal (Biorad, Hercules, CA, USA) was measured using the LI-COR Odyssey Imaging System and analyzed with Image Studio™ software (LI-COR Biosciences, Bad Homburg, Germany). Equal secreting cell lines were selected for in vivo injection.

### 4.12. Mice 

Six to twelve-week-old female C57BL/6 mice were purchased from Charles River. All animals were handled according to the institutional guidelines. C57BL/6 *Nrp-1*^FL/FL^ (NRP-1^High^) were a gift from Dr. Gu (Harvard University). LysM^Cre^*Nrp-1*^FL/FL^ (NRP-1^Low^) were generated by intercrossing *Nrp-1* floxed mice with LysM-Cre mice (the Jackson Laboratory) and previously described by Casazza et al. [5]. Experiments were approved by the Ethical Committee for use of laboratory animals of the Vrije Universiteit Brussel (VUB) (ECD n° 16-214-4, 18-214-10 and 18-214-12). 

### 4.13. Tumor Challenge

C57BL/6 mice received a subcutaneous injection at the tail base of 3 × 10^5^ (in 50 µL PBS) MC38 cells that produced BCII10 or Nb1. When indicated, mice received an intraperitoneal injection of mAbs (50 µg in 50 µL PBS), either a control (Clone LTF-2, BioXCell) or CD8-depleting antibody (Clone 2.43, BioXCell, TE Huissen, The Netherlands). Since depletion of CD8^+^ T cells is maintained for 3–4 days, the intraperitoneal injection of antibodies was repeated every third day. The tumor volume was measured thrice a week using an electronic caliper. The tumor volume was calculated using the following formula: (length × width^2^)/2.

### 4.14. Preparation of a Single-Cell Suspension from Tumors 

Tumors were isolated at the indicated size and single-cell suspensions were prepared using the GentleMACS single-cell isolation protocol (Miltenyi Biotec, Bergisch Gladbach, Germany). Briefly, tumors were isolated and minced into small pieces, followed by a mechanical dissociation step using the GentleMACS dissociator. Samples were then incubated for 40 min at 37 °C in 5 mL RPMI1640 containing the following enzymes: 150 µL collagenase I (10,000 U/mL, Sigma-Aldrich), 150 µL dispase II (32 mg/mL, Sigma-Aldrich) and DNase I (10 U/mL, Sigma-Aldrich). After a final mechanical disruption step, the digested tumors were harvested, filtered over a 70 μm nylon filter and red blood cells were lysed using Tris-buffered ammonium chloride. The single-cell suspensions for flow cytometry analysis were washed and stained in PBS containing 1% BSA and 0.02% sodium azide. 

The single-cell suspensions for the macrophage sorting were washed and stained in MACS buffer (0.5% FBS, 2mM EDTA) with a final concentration of 1 × 10^8^ cells/mL in round-bottom tubes.

### 4.15. Polymerase Chain Reaction (PCR)

RNA was extracted using the RNeasy mini kit according to manufacturer’s protocol (Qiagen). The total RNA fraction was converted to cDNA using the Verso™ cDNA Synthesis kit (Thermo Fisher Scientific). Expression of BCII10, Nb1 and actin by MC38 cells was evaluated using the KAPA2G Robust HotStart PCR mix (Merck, Darmstadt, Germany) using the primers listed in Table 1. PCR products were analyzed on a 1.2% agarose gel. Gene expression analysis of macrophages sorted from tumors was performed using a CFX96™ Real-Time PCR detection system (Biorad, Hercules, CA, United States). Each PCR reaction contained 5 µL iQ SYBR™ Green Supermix (Bio-Rad), 250 nM of each primer and 20 ng template DNA in water to a total volume of 10 µL. Each PCR reaction was carried out with an initial incubation of 95 °C for 3 min followed by 40 cycles of denaturation at 95 °C for 10 s and combined annealing and extension at 55 °C for 30 s. The target gene expression was calculated using the comparative threshold method and was normalized to *hprt1*.

### 4.16. Flow Cytometry and Fluorescence-Assisted Cell Sorting

Flow cytometry was performed to analyze (1) expression of NRP-1 and binding of Nbs to NRP-1 on PEMs, (2) the phenotype and transgene expression of in vitro cultured cells or (3) the phenotype of cells derived from tumors. The first step of each staining was performed in the presence of 5% normal goat serum (Sigma-Aldrich) and 1/50 anti-CD16/32 antibody (clone 93, BioLegend, San Diego CA, USA) to reduce non-specific antibody binding. Stainings were performed for 30 min on ice in PBS containing 1% BSA and 0.02% sodium azide (FACS buffer) unless otherwise described. 

PEMs were incubated with NRP-1-specific Nbs for 30 min in PBS containing 0.2% BSA, followed by staining with phycoerythrine (PE)-conjugated anti-HIS antibodies (R&D systems) for 30 min. 

HEK293T and MC38 cells that express Nbs were permeabilized in 100 µL Cytofix/Cytoperm solution (BD Biosciences) at 4 °C for 10 min and washed with Perm/Wash buffer (BD Biosciences), followed by intracellular staining with PE-conjugated anti-HA antibodies (Abcam) at 4 °C for 25 min. Analyses were performed on 10,000 viable single cells for in vitro cultivated cells and on 50,000 viable single cells when derived from in vivo grown tumors as determined by light scatter properties using FACSDiva software on an LSR Fortessa (Becton Dickinson, Franklin Lakes, NJ, USA).

Tumor-derived cells were stained with antibodies for the detection of several myeloid and T cell markers and for the sorting of macrophages from the TME. The allophycocyanin cyanin7-conjugated antibody specific for CD45 (clone 104), the AF700-conjugated antibody specific for CD11b (clone M1/70) and CD4 (clone RM4-5), PE-Cy7-conjugated antibody specific for Ly6C (clone HK1.4) the AF647-conjugated antibody specific for Ly6G (clone 1A8) and CD19 (clone AD3) and PE/Dazzle594-conjugated antibody specific for MHC-II (clone M5/114.15.2) were purchased from BD Biosciences. The allophycocyanin-conjugated dextramer specific for Reps1 peptide (AQLANDVVL) presented by H2-D^b^ was purchased from Immudex. The PerCP-Cy5.5-conjugated antibody specific for F4/80 (clone BM8) and CD127 (clone A7R34), the AF488-conjugated antibody specific for CD11c (clone N418), PE-Cy7-conjugated antibody specific for CD3 (clone 17A2) and the PE-conjugated antibody specific for CD206 (clone C068C2) were purchased from BioLegend. Analyses were performed on viable cells as determined by light scatter properties using FACSDiva software on a LSR Fortessa (Becton Dickinson). Macrophages (50,000 cells) were sorted from the TME with a FACSAria II (BD Biosciences). Sorted macrophages were collected in 15 mL polystyrene tubes pre-coated with FBS containing 2 mL RPMI-1640 medium (Sigma-Aldrich) supplemented with 20% FBS.

### 4.17. Statistical Analyses

Statistical analysis was performed by the Student’s *t*-test using GraphPad Prism 7.0 software. Sample sizes were calculated to determine the minimal needed number of animals using G*Power statistical analysis software developed by Faul et al. [58]. Sample sizes and the number of times experiments were repeated are indicated in the figure legends. Survival curves were compared using the log-rank (Mantel–Cox) test and Gehan–Breslow–Wilcoxin test. The number of asterisks in the figures indicates the level of statistical significance, as follows: * for *p* < 0.05; ** for *p* < 0.01, *** for *p* < 0.001 and ns for non-significant. The results are shown in a column graph as the mean ± SEM.

## 5. Conclusions

In conclusion, our data demonstrate that targeting NRP-1 in cancer, using antagonistic anti-NRP-1 Nbs that prevent the interaction with Sema3A, delays growth of CRC tumors and extends survival through a shift in the MHC-II^high^/MHC-II^low^ ratio and an activation of CRC-specific CD8^+^ T cells. These findings provide a rationale for the further development of Nbs targeting human NRP-1 and bringing them from the bench to the bedside.

## Figures and Tables

**Table 1:** Primers used in RT-PCR or RT-qPCR.

*RT-PCR*	Forward	Reverse
***Actin***	5′-CTG TCC CTG TAT GCC TCT G-3′	5′-ATG TCA CGC ACG ATT TCC-3′
***BCII10***	5′-TCC TGC TAT GGG TAC TGC TGC T-3′	5′-CTC AGG TTT CAG GTT GTT CAT TT-3′
***Nb1***	5′-TCC TGC TAT GGG TAC TGC TGC T-3′	5′-GTA ATC TTT GCG ACC AAC TCG-3′
*Itgam* (CD11b)	5′-TCT TGG GTT TCC TAG TGT GTT AG-3′	5′-AGA GGA CAG CAC AGC ATT TAG-3′
*Adgre1* (F4/80)	5′-CGT CAG GTA CGG GAT GAA TAT AAG-3′	5′-ATC TTG GAA GTG GAT GGC ATA G-3’
*H2* (MHC-II)	5’-CAG CAA GGA CTG GTC TTT CTA T-3′	5′-AAC TCT GCA GGC GTA TGT ATC-3′
*Tnf* (Tnfα)	5′-CCT TCA CAG AGC AAT GAC TC-3′	5′-GTC TAC TCC CAG GTT CTC TTC-3′
*Ifng* (Ifnγ)	5′-CGG CAC AGT CAT TGA AAG CCT A-3′	5′- GTT GCT GAT GGC CTG ATT GTC-3′
*Il1b*	5′-GTG TGG ATC CAA AGC AAT AC-3′	5′-GTC TGC TCA TTC ATG ACA AG-3′
*Il12b*	5′- GAA AGA CCC TGA CCA TCA CT-3′	5′-CCT TCT CTG CAG ACA GAG AC-3′
*Mrc1* (CD206)	5′-GCA AAT GGA GCC GTC TGT GC-3′	5′-CTC GTG GAT CTC CGT GAC AC-3′
*Arg1*	5′-GTC CCT AAT GAC AGC TCC TTT C-3′	5′-CCA CAC TGA CTC TTC CAT TCT T-3′
*Ptgs2* (Cox2)	5′-CAG ACA ACA TAA ACT GCG CCTT 3′	5′-GAT ACA CCT CTC CAC CAA TGA CC 3′
*Il10*	5′-ACT CAA TAC ACA CTG CAG GTG-3′	5′-GGA CTT TAA GGG TTA CTT GG-3′
*Hif1a*	5′-ACC TGG CAA TGT CTC CTT TAC-3′	5′-CCA GTG ACT CTG GAC TTG ATT C-3′
*Stab1*	5′-ACG GGA AAC TGC TTG ATG TC-3′	5′-ACT CAG CGT CAT GTT GTC CA-3′
*Chil3* (*Ym1*)	5′-GCT AAG GAC AGG CCA ATA GAA-3′	5′-GCA TTC CAG CAA AGG CAT AG-3′
*Lyve1*	5′-CTG GCT GTT TGC TAC GTG AA-3′	5′-CAT GAA ACT TGC CTC GTG TG-3′
*vegfa*	5′-CAC TTC CAG AAA CAC GAC AAA C-3′	5′-TGG AAC CGG CAT CTT TAT CTC-3′
*vegfr2*	5′-CTC TGT CAA GTG GCG GTA AA-3′	5′-TCA GGA AGC CAC AAA GCT AAA -3′
*Hprt1*	5′-CGA GAT GTC ATG AAG GAG ATG G-3′	5′-AGC AGG TCA GCA AAG AAC TTA-3′

## Supplementary Materials

The following are available online at https://www.mdpi.com/2072-6694/12/12/3582/s1, Figure S1: Gating strategy to define T cells in flow cytometry, Figure S2: Gating strategy to define myeloid cells in flow cytometry, Figure S3: Local production of Nb1 does not significantly affect myeloid cell numbers, Figure S4: Gating strategy to define Treg cells in flow cytometry, Figure S5: Nb1 can reduce migration of BMDMs towards Sema3A, Figure S6: Full western blot images for Figure 4B, Figure S7: Full western blot images for Figure 4B.
