# Supplementary material for: Targeting Neuropilin-1 with Nanobodies Reduces Colorectal Carcinoma Development
Source: Cancers (Basel). 2020 Nov 30;12(12):3582. doi: 10.3390/cancers12123582 (PMC7760077; doi:10.3390/cancers12123582)
Supplement: Supplementary file 1 [file cancers-12-03582-s001.pdf]

Supplementary Materials: Targeting Neuropilin-1 With Nanobodies Reduces Colorectal Carcinoma Development

Yannick De Vlaeminck, Stefano Bonelli, Robin Maximilian Awad, Maarten Dewilde, Sabrina Rizzolio, Quentin Lecocq, Evangelia Bolli, Ana Rita Santos, Damya Laoui, Steve Schoonooghe, Luca Tamagnone, Cleo Goyvaerts, Massimiliano Mazzone, Karine Breckpot and Jo A. Van Ginderachter

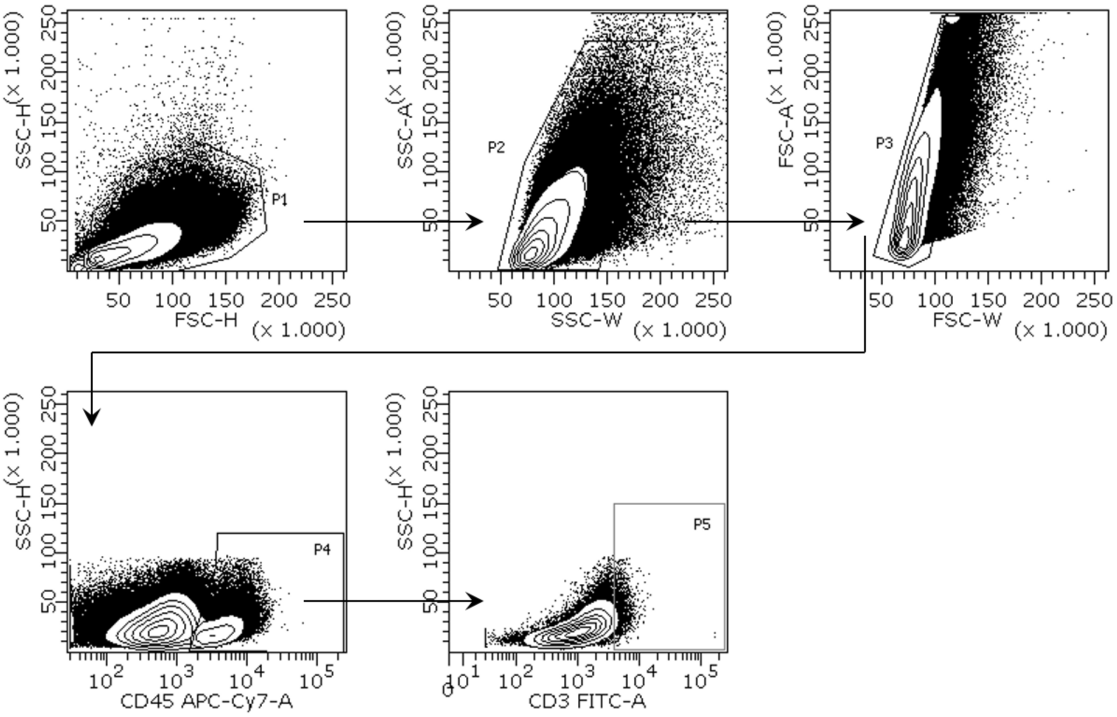

Figure S1. Gating strategy to define T cells in flow cytometry. MC38 tumors were grown at the lower back of C57BL/6 mice up to a volume of 944±237mm³, after which tumors were isolated and reduced to single-cell suspensions. Expression of NRP-1 was evaluated on tumor-infiltrating immune cells and non-immune cells in flow cytometry. The flow cytometry density graphs depict the gating strategy that was employed to define T cells. Herein, single viable cells were first gated based on forward scatter (FSC) and side scatter (SSC) characteristics. Within the gate of viable singlets, $CD45^+$ versus $CD45^-$ cells were visualized, after which the $CD3^+$ cell population was determined within the $CD45^+$ fraction. Gates were based on control samples that were stained with the CD45-specific antibody, however not with the CD3-specific antibody. The data are a representative of two independent experiments (n = 2, mpc = 3).

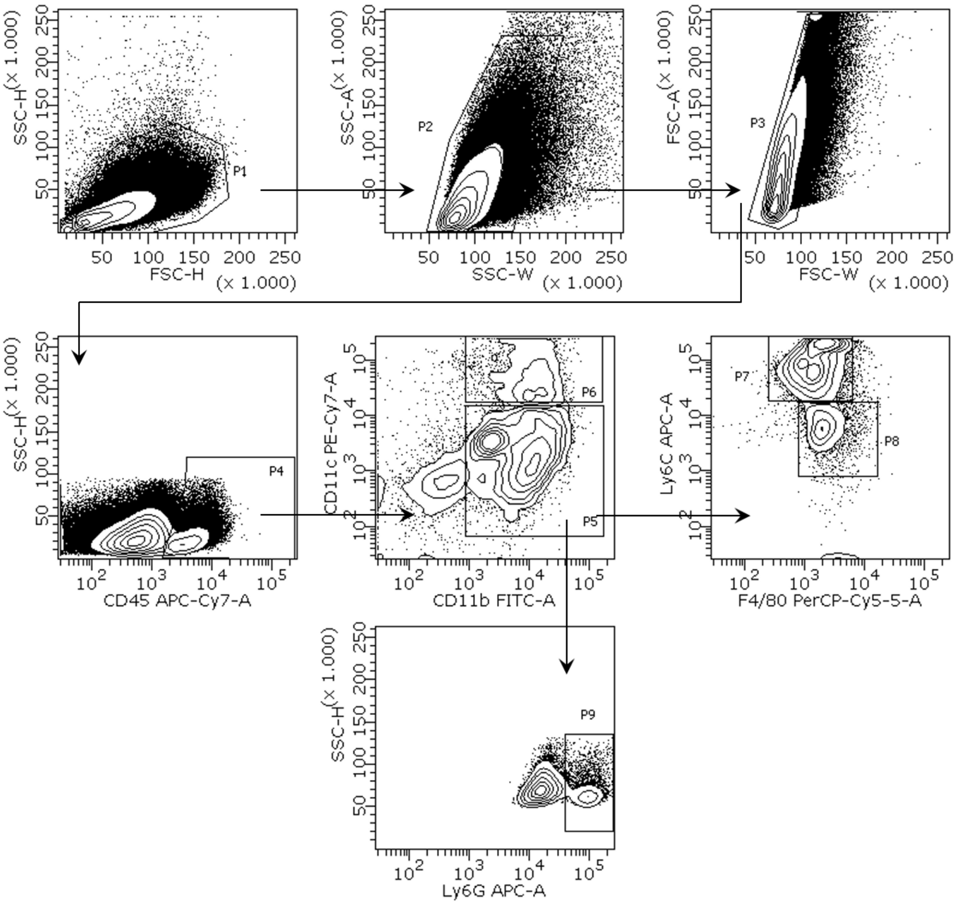

Figure S2. Gating strategy to define myeloid cells in flow cytometry. MC38 tumors were grown at the lower back of C57BL/6 mice up to a volume of 944±237mm^3, after which tumors were isolated and reduced to single-cell suspensions. Expression of NRP-1 was evaluated on tumor-infiltrating immune cells and non-immune cells in flow cytometry. The flow cytometry density graphs depict the gating strategy that was employed to define dendritic cells (DCs), monocytes, macrophages and granulocytes. Single viable cells were first gated based on FSC and SSC characteristics. Within the gate of viable singlets, CD45$^+$ versus CD45$^-$ cells were visualized, after which the CD11b$^+$ and CD11c$^+$ (DC) populations were determined within the CD45$^+$ fraction. The CD11b$^+$ population was further subdivided according to expression of the cell surface markers Ly6C (monocytes), F4/80 (macrophages) and Ly6G (neutrophils). The data are a representative of two independent experiments (n = 2, mpc = 3).

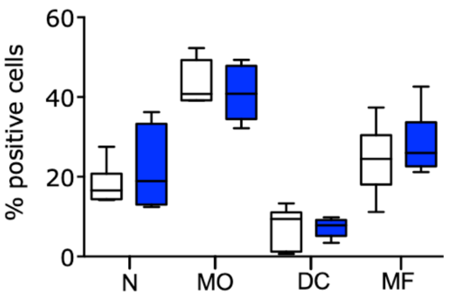

Figure S3. Local production of Nb1 does not significantly affect myeloid cell numbers. MC38/BCII10 or MC38/Nb1 were grown at the lower back of C57BL/6 mice. Growth of MC38 tumors was monitored on a daily basis. The box and whisker graphs show the range, mean and standard deviation of myeloid cell numbers in the TME. Neutrophils (N), monocytes (MO), dendritic cells (DC) and macrophages (MF) were defined based on the gating strategy shown in Supplementary Figure 2.

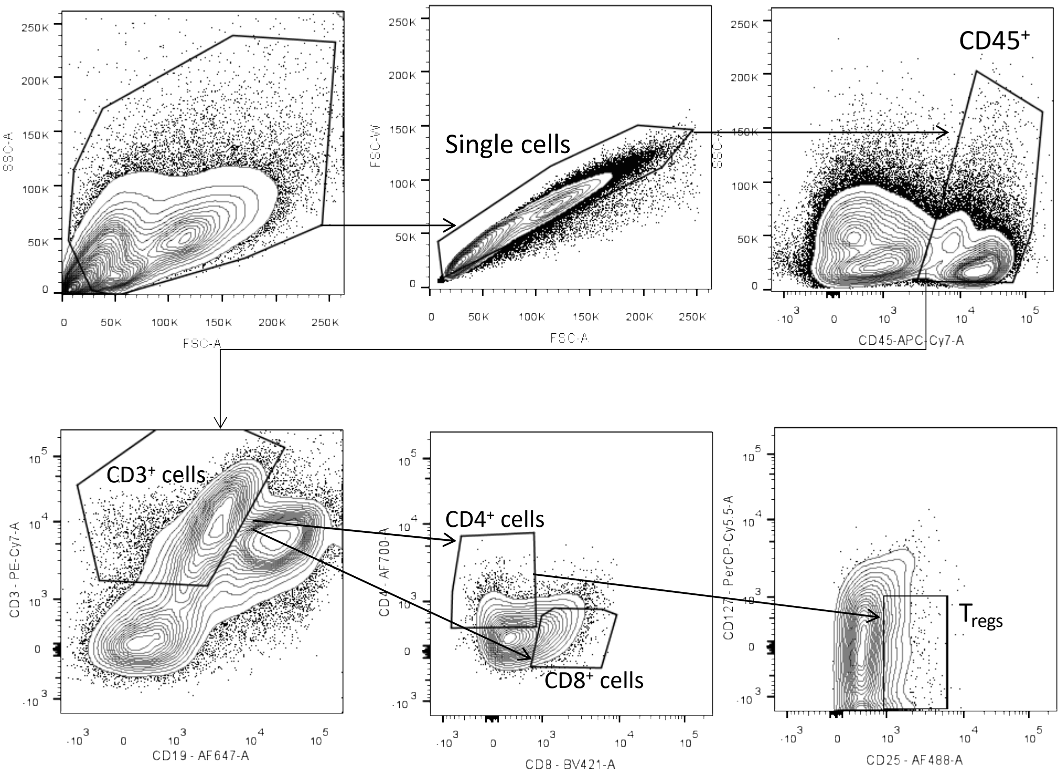

Figure S4. Gating strategy to define Treg cells in flow cytometry. MC38 tumors were grown at the lower back of C57BL/6 mice up to a volume of 534±68mm³, after which tumors were isolated and reduced to single-cell suspensions. The flow cytometry density graphs depict the gating strategy that was employed to define Tregs. Herein, single viable cells were first gated based on FSC and SSC characteristics. Within the gate of viable singlets, CD45⁺ versus CD45⁻ cells were visualized, after which the CD3⁺/CD19⁻ cell population was determined within the CD45⁺ fraction. Within the CD4⁺ population, Tregs were defined as CD25^{high}/CD127⁻. The data are a representative of one experiment (mpc = 10).

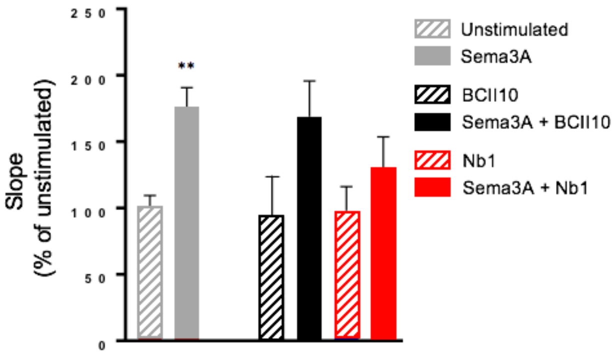

Figure S5. Nb1 can reduce migration of bone marrow-derived macrophages (BMDMs) towards Sema3A. The migration of fluorescently labeled BMDMs seeded in a trans-well system were monitored in time in the presence of control (BCII10) or NRP-1-blocking Nbs (Nb1) with or without Sema3A. The percent slope of the fluorescence units was calculated for each condition and compared to the slope of unstimulated BMDMs (n = 1).

Agarose gel 1.2 % (Figure 4.B)

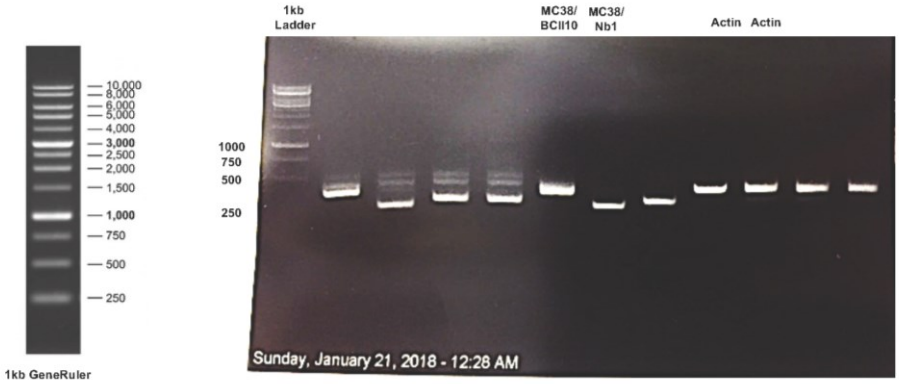

Figure S6. Full western blot images for Figure 4B.

Western Blot (Figure 4.D)

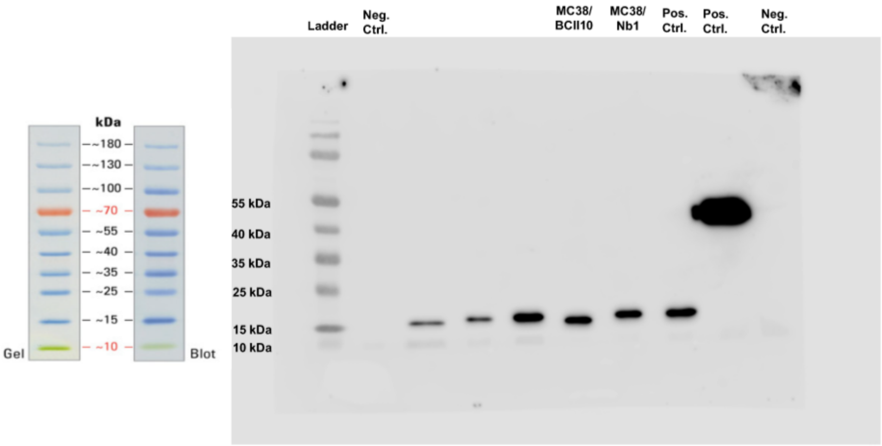

Figure S7. Full western blot images for Figure 4B.
